# The role of neurotrophin genes involved in the vulnerability to gambling disorder

**DOI:** 10.1038/s41598-022-10391-w

**Published:** 2022-04-28

**Authors:** Neus Solé-Morata, Isabel Baenas, Mikel Etxandi, Roser Granero, Sonia V. Forcales, Manel Gené, Carme Barrot, Mónica Gómez-Peña, José M. Menchón, Nicolás Ramoz, Philip Gorwood, Fernando Fernández-Aranda, Susana Jiménez-Murcia

**Affiliations:** 1 Department of Psychiatry, Bellvitge University Hospital, c/Feixa Llarga S/N, Hospitalet de Llobregat, 08907 Barcelona, Spain; 2 Psychoneurobiology of Eating and Addictive Behaviors Group, Neurosciences Program, Bellvitge Biomedical Research Institute (IDIBELL), Hospitalet de Llobregat, Spain; 3 Ciber Physiopathology of Obesity and Nutrition (CIBERObn), Instituto de Salud Carlos III, Barcelona, Spain; 4 Department of Psychobiology and Methodology, Autonomous University of Barcelona, Bellaterra, Spain; 5 Serra Húnter Programme, Department of Pathology and Experimental Therapeutics, Faculty of Medicine and Health Sciences, University of Barcelona, Hospitalet de Llobregat, 08907 Spain; 6 Genetic Lab, Forensic and Legal Medicine Unit, Department of Clinical Sciences, School of Medicine and Health Sciences, University of Barcelona, Barcelona, Spain; 7 Department of Clinical Sciences, School of Medicine and Health Sciences, University of Barcelona, Hospitalet del Llobregat, Spain; 8 Ciber of Mental Health (CIBERSAM), Instituto de Salud Carlos III, Barcelona, Spain; 9 Psychiatry and Mental Health Group, Neuroscience Program, Bellvitge Biomedical Research Institute (IDIBELL), Hospitalet del Llobregat, Spain; 10 Institute of Psychiatry and Neuroscience of Paris (IPNP), INSERM U1266, Team Vulnerability of Psychiatric and Addictive Disorders, Université de Paris, 75014 Paris, France

**Keywords:** Predictive markers, Addiction

## Abstract

Evidence about the involvement of genetic factors in the development of gambling disorder (GD) has been assessed. Among studies assessing heritability and biological vulnerability for GD, neurotrophin (NTF) genes have emerged as promising targets, since a growing literature showed a possible link between NTF and addiction-related disorders. Thus, we aimed to explore the role of NTF genes and GD with the hypothesis that some NTF gene polymorphisms could constitute biological risk factors. The sample included 166 patients with GD and 191 healthy controls. 36 single nucleotide polymorphisms (SNPs) from NTFs (NGF, NGFR, NTRK1, BDNF, NTRK2, NTF3, NTRK3, NTF4, CNTF and CNTFR) were selected and genotyped. Linkage disequilibrium (LD) and haplotype constructions were analyzed, in relationship with the presence of GD. Finally, regulatory elements overlapping the identified SNPs variants associated with GD were searched. The between groups comparisons of allele frequencies indicated that 6 SNPs were potentially associated with GD. Single and multiple-marker analyses showed a strong association between both NTF3 and NTRK2 genes, and GD. The present study supports the involvement of the NTF family in the aetiopathogenesis of GD. An altered cross-regulation of different NTF members signalling pathways might be considered as a biological vulnerability factor for GD.

## Introduction

Gambling is an increasingly activity in our society, especially in online modality^1^. Although many people gamble without undergoing health problems, some individuals develop gambling disorder (GD), leading to severe psychological, social and economic consequences^2^. Among adults, lifetime prevalence of GD range between 0.02 and 2.0%^3^. Similar values have been reported in Europe, where the prevalence of GD fluctuates between 0.3 and 3.0%^4–6^. Gambling disorder was first recognized as a mental disorder by the American Psychiatric Association in the third edition of the Diagnostic and Statistical Manual of Mental Disorders (DSM-III)^7^. Although GD was initially classified as an impulse control-disorder (ICD), it shares similarities with substance addictions and it has been reclassified as an addictive disorder in the fifth edition of the DSM (DSM-5)^8–10^. Nevertheless, some researchers’ opinion favors leaving GD in the ICD category^11^.

Several studies showed that certain individuals may present an increased risk for GD: for example, male gender, lower socio-economic status, early gambling onset, high impulsivity and sensation seeking are some of the factors that have been repeatedly associated with GD^12^. Although the aetiology of GD is not fully understood, it has to be seen as a complex interrelation between psychological, social and biological factors^13^. Over the last years, multiple aetiological models for GD have been proposed, such as the cognitive-behavioural model^14^ or the biopsychosocial model^15^*.* One of the most prominent model, the three pathways model of GD, recognized the multidimensional nature of the disorder and claimed that pathological gamblers form an heterogenous population^16^. This model suggested the existence of three different groups of individuals with GD (behaviourally conditioned, emotionally vulnerable and antisocial impulsivist), with each group exhibiting its own gambling-related motivations. For example, individuals in the antisocial impulsivist group tend to gamble for positive-reinforcement motivations (such as sensation-seeking)^16,17^.

From a neurobiological perspective, several studies have described similarities between neurochemical profiles and brain structures among patients with substance-related addictive disorders and behavioural addictions^18,19^. For instance, neuroimaging studies have suggested that abnormalities in the ventral striatum could contribute to impulsive behaviours in individuals with GD^20–22^. Similarly, coincidences regarding the neurobiological mechanisms (such as neurotransmitters) underlying impulsivity, compulsivity, reward/withdrawal and decision making have also been described^11^. For example, a key mechanism involved in motivation and reward processing is the dopaminergic system^23^. Indeed, increased dopamine transmission in the dorsal striatum has also been linked to severity of problem gambling^24^. Molecular genetic studies of GD applying candidate gene approach have also suggested the possible involvement of dopaminergic (such as DRD1, DRD2 and DRD4) and serotonergic (such as SLC6A4, MAOA and MAOB) genes in conferring vulnerability for GD^18,25^.

Recently, Genome Wide Association Studies (GWAS) have made a considerable progress towards an understanding of many complex diseases^26^. However, to date, only two GWAS of GD have been reported. First, Lind et al.^27^ performed a GWAS for disordered gambling using a quantitative factor score in 1312 twins from 894 Australian families. Despite no single-nucleotide polymorphism was associated with the disorder, using a less-stringent threshold the authors reported six variants in three genes (MT1X, ATXN1 and VLDLR) associated with GD, that had been previously linked to addiction and other psychiatric disorders^27^. A few years later, Lang et al.^28^ conducted the first and only case–control GWAS of GD, with all cases being assigned a diagnosis of GD. Despite this study did not identify genome-wide significant regions for GD, an association between a polygenic risk score for alcoholism and severity of problem gambling was found, supporting a link between alcohol use and GD^28^. Finally, a recent study has examined the relationship between a Parkinson's disease (PD) polygenic risk score (PRS) and impulse control disorders (ICDs) in PD. Although the results showed no association between the 90 SNPs analyzed and the prevalence of ICDs, a younger disease onset was associated with both higher PRS and ICD prevalence^29^. That being said, genetic studies using larger sample sizes are needed. Furthermore, global *omic* approaches that interrogate epigenetic differences could complement genetic studies, linking both events to the identification of novel altered gene regulatory mechanisms implicated in GD.

Furthermore, because of their involvement in the regulation of activity-dependent neuronal function and synaptic plasticity, neurotrophic factors (NTF) have been linked with the pathophysiology of several neuropsychiatric disorders, such as addictions^30–33^. The NTF family comprises four related proteins, namely nerve growth factor (NGFB), brain-derived neurotrophic factor (BDNF) and neurotrophins 3 and 4/5 (NTF3 and NTF4/5). The effects of NTFs are mediated by high affinity tyrosine kinase receptors (NTRK), each preferentially activated by one or more NTF: NTRK1 by NGFB, NTRK2 by BDNF and NTF4/5, and NTRK3 by NTF3. Moreover, a low-affinity pan-NTF receptor (p75), a member of the tumoral necrosis factor family, forms a complex with other NTF receptors and modulates its signal transduction^34,35^.

In particular, BDNF and its genes constitute one of the most studied elements among addiction-related disorders^30,32,33^. It has been suggested that drugs of abuse induce neuroadaptative processes in the brain, and these changes involve regulation of BDNF activity^30^. However, the exact role of BDNF and other NTFs on the aetiopathogenesis of addictive disorders is still unknown. In the case of GD, only a few studies have examined its association with NTFs, which reported an increase of mean serum BDNF levels in patients with GD compared to healthy controls, suggesting a link between BDNF levels and GD severity^36–39^. Therefore, as described in other addictions, the link between NTFs and GD could be a mutual process: the addictive behaviour might affect the expression of NTFs and NTFs can modulate the addictive behaviour^30^. Nevertheless, further work needs to be performed to establish the exact role of these genes on the GD clinical profile.

Therefore, the main aim of the present study was to explore the genetic relationship between NTF and GD. We hypothesized that some NTF genes polymorphisms could constitute potential biological risk factors due to their implication in the onset and progression of GD. Hence, we selected informative polymorphisms spanning the entire loci of p75 (NGFR), NGFB, NTRK1, BDNF, NTRK2, NTF3, NTRK3, NTF4, CNTF and CNTFR. Then, we analysed the variation within each gene comparing a clinical group of patients with GD and a group of healthy controls.

## Results

### Single markers of GD

Starting from the 158 SNPs measured in the study, a total of 36 SNPs were pre-selected for the haplotype analysis based on its capacity to discriminate the presence of GD in univariate tests (χ^2^) or multivariate tests (stepwise logistic regression). This initial selection was done considering different genetic models for each SNP (codominant, dominant, recessive, overdominant and log-additive), and a SNP was selected for the analysis in Haploview when *p* < 0.05 was obtained in any of the analyses.

**Figure 1:**
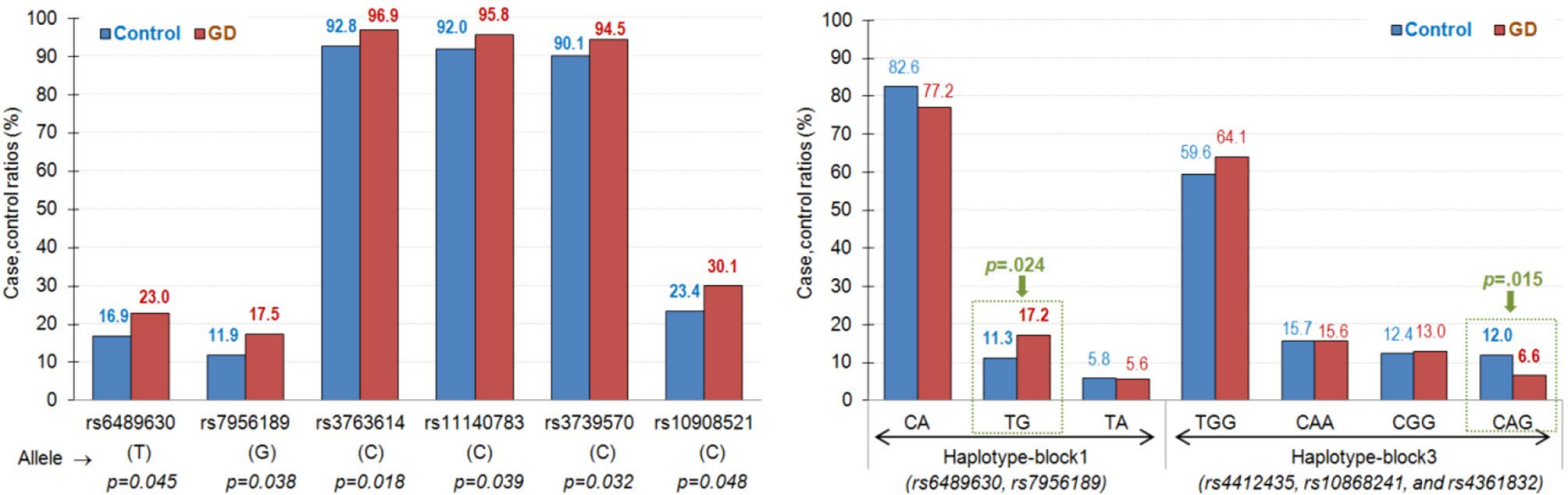
Single markers (first panel) and haplotype blocks (second panel) associated to the presence of GD. Adjusted p-values for correcting increase in Type-I error due to multiple null-hypothesis tests.

Table S1 (supplementary material) includes the basic information for the 36 SNPs selected in this study (chromosome, gene, and allele), as well as the results obtained in Haploview regarding the conformance with HWE, the distribution of the allele frequency into the groups and the association tests for single markers. All the HWE tests achieved *p* > 0.05 in our study. The comparison of the allele frequency distribution between the groups indicated that the 6 SNPs were potentially associated with the presence of GD (see first panel of Fig. 1): rs6489630 (T allele), rs7956189 (G allele), rs3763614 (C allele), rs11140783 (C allele), rs3739570 (C allele) and rs10908521 (C allele).

**Figure 2:**
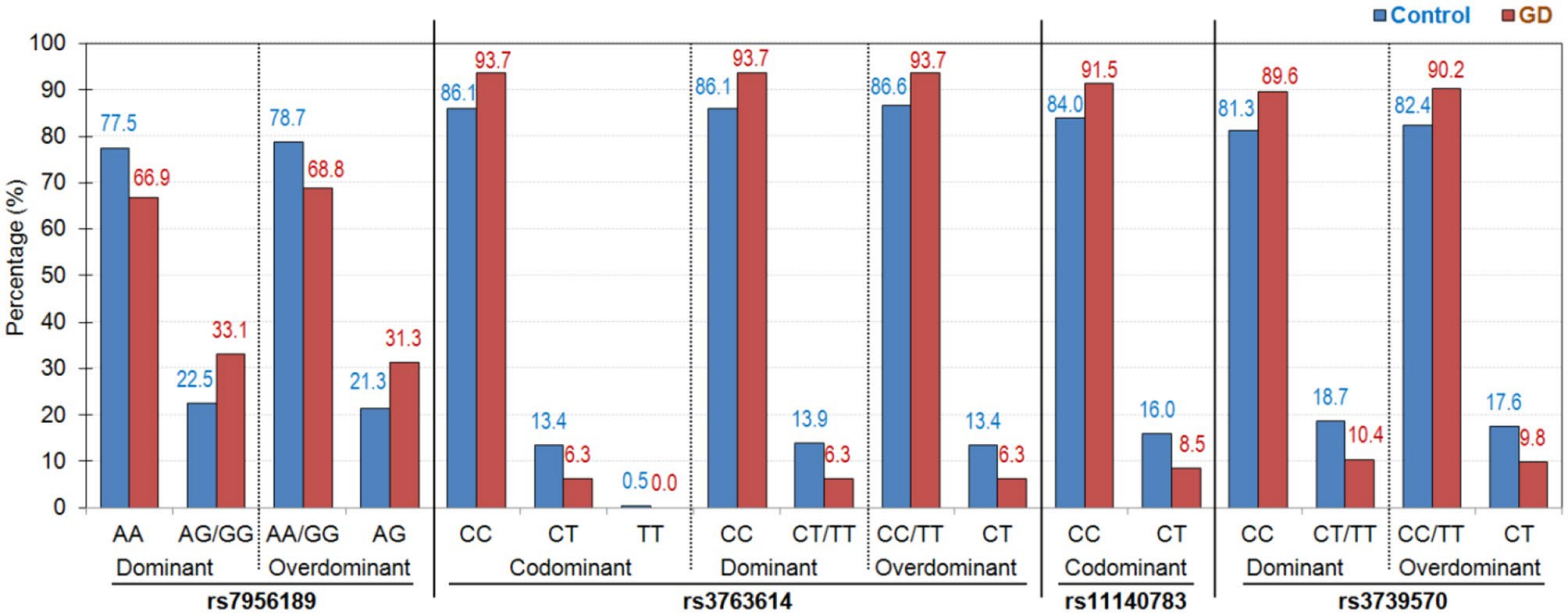
Single nucleotide polymorphism under different genetic models achieving risk for the presence of GD.

Figure 2 shows the results to assess the link between the 6 SNPs associated to the presence of GD according to different genetic models (also see supplementary Table S2, which includes the complete frequency distribution and the p-values obtained in logistic regression models adjusted by the covariates sex, age, education and employment status). These analyses confirmed that 4 single SNPs were significantly related to GD (*p* < 0.05) with an increase in the risk of GD for: (a) rs796189, the presence of genotype “AG/GG” (dominant model) and “AG” (overdominant model) and; (b) rs3763614, the presence of genotype “CC” (codominant and dominant models) and genotype “CC/TT” (overdominant model); (c) rs11140783, the presence of genotype “CC” (codominant model); and d) rs3739570 the presence of genotype “CC” (dominant model) and “CC/TT” (overdominant model). Quasi-significant results (*p* < 0.10) were achieved for the SNPs rs6489630 (dominant model with genotype “CC” related to higher risk for GD) and rs10908521 (codominant and recessive models with higher risk of GD associated with genotype “CC”).

### Haplotype analysis

**Figure 3:**
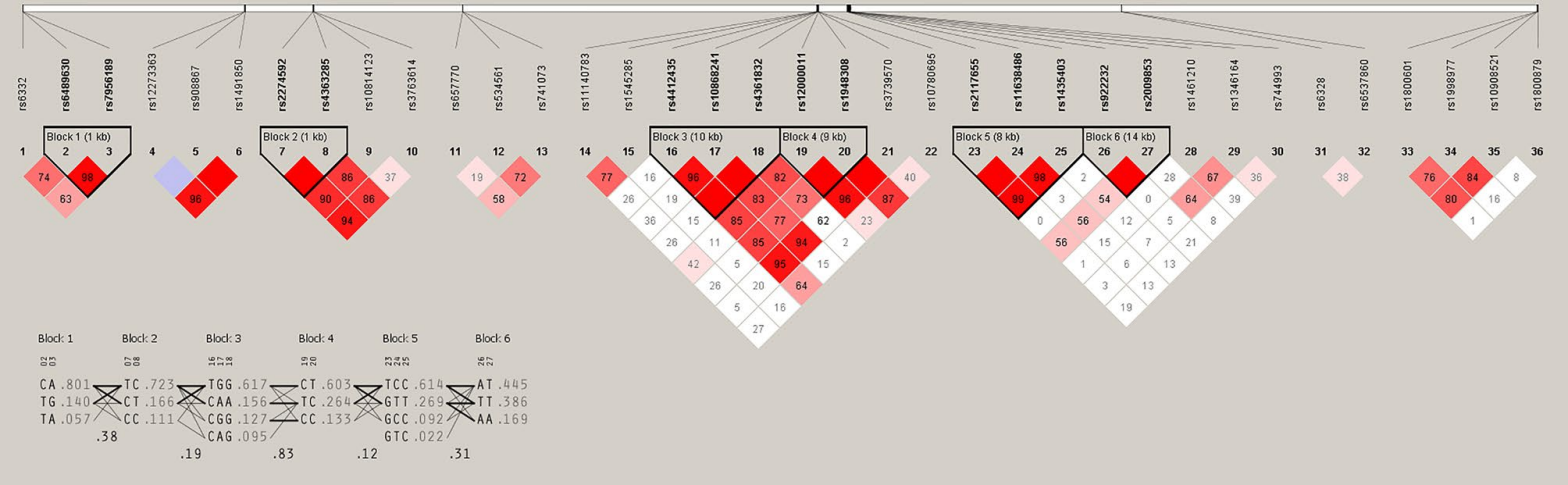
Haplotype block map. The upper part of the figure shows the haplotypes block map and the lower part contains the transition scheme of haplotypes. The lines in the transition image show the movement from one block to the next. The frequency corresponds to the thickness of the line (connections with thin lines if higher than 1% and with thick lines if higher than 10%), and the D′ coefficient measures the degree of linkage disequilibrium (LD) between the blocks.

Six LD blocks were identified in the study (the upper part of Fig. 3 shows the haplotypes block map). Block 1 included SNPs rs6489630 and rs7956189 in NTF3 gene, and haplotype “TG” was significantly related to an increase in the risk of GD (*p* = 0.045) (Table S3 and Fig. 3). Block 3 included the SNPs rs4412435, rs10868241, and rs4361832 in NTRK2 gene, and haplotype CAG (*p* = 0.048) was related to a decreased risk of GD. Other SNPs included in the blocks were: rs2274592 and rs4363285 (inside block 2) among CNTFR gene, rs12000011 and rs1948308 (inside block 4) in NTRK2 gene, rs211765, rs11638486 and rs1435403 (inside block 5), and rs922232 and rs2009853 (inside block 6), both in NTRK3 gene. No significant haplotype in the blocks 2, 4, 5 and 6 were significantly related to an increase/decrease in the risk of GD.

The lower part of Fig. 3 contains the transition scheme of haplotypes. The lines in the transition image show the movement from one block to the next. The frequency corresponds to the thickness of the line (connections with thin lines if higher than 1% and with thick lines if higher than 10%), and the *D’* coefficient measures the degree of LD between the blocks.

**Figure 4:**
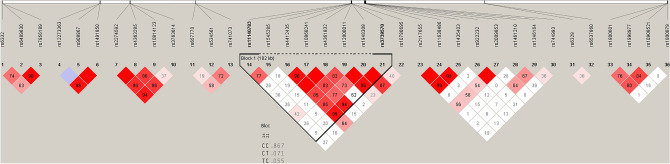
Haplotype block map of block 7 (defined manually).

Finally, a new LD block was manually defined by the SNPs rs11140783 and rs3739570 (Table S3 and Fig. 4) among NTRK2 gene, showing a significant association of haplotype CC with an increase in the risk of GD (*p* = 0.012).

### Overlap with putative regulatory regions

Most of the genetic variants pre-selected for the haplotype analysis are located at non-coding regions of the human genome, with predominant sites being introns (see Table 1). Gene regulatory regions, such as enhancers and promoters are usually located upstream of genes and in 5′UTRs. However intron-bearing transcriptional factors (TF) binding sites have also been revealed by several studies^40^. Moreover, chromatin capture data shows that distant genomic loci, which can be kilobases, and megabases apart, interact to regulate gene transcription and to maintain genomic boundaries, which in turn sustain a particular chromatin architecture. Therefore, an association of these SNPs with regulatory regions or three-dimensional (3D) contact loci could uncover a functional task for these variants, ranging from transcriptional control, mRNA stability to chromatin dynamics. It reveals an unprecedented impact in GD pathophysiology.

**Table 1:** Genomic annotation of variants.

SNP	Gene	Genomic region	ENCODE Cis regulatory elements (CREs)	TF binding sites (JASPAR)
rs6332	NTF3	Synonymous variant	–	–
rs6489630	NTF3	Downstream gene variant	pELS (EH38E1589222)	–
rs7956189	NTF3	Downstream gene variant	–	WT1
rs12273363	BDNF	Upstream gene variant	pELS (EH38E1528854)	YY1
rs908867	BDNF	Upstream gene variant	–	–
rs1491850	BDNF	Intron variant	–	–
rs2274592	CNTFR	Intron variant	–	ZNF740, WT1, CTCF, Znf281
rs4363285	CNTFR	Intron variant	–	ELF3, SPIB, ELF1, EHF, CTCF
rs10814123	CNTFR	Intron variant	–	HFN1A, BARX2
rs3763614	CNTFR	Intron variant	–	Nr5a2
rs657770	NGFR	Intron variant	–	–
rs534561	NGFR (P75)	Intron variant	–	MZF1(var.2)
rs741073	NGFR (P75)	3 prime UTR variant	adjacent to a dELS (EH38E1868997)	KLF9, MSANTD3
rs11140783	NTRK2	Intron variant	Adjacent to a dELS (EH38E2702614)	IRF8, IRF4
rs1545285	NTRK2	Intron variant	–	Dmbx1, OTX2
rs4412435	NTRK2	Intron variant	dELS (EH38E2702692)	BACH2, NFE2L1, MAFK, JUN (var.2), MAF::NFE2, Smad2::Smad3
rs10868241	NTRK2	Intron variant	–	Prmd15
rs4361832	NTRK2	Intron variant	–	NFIA, MSGN1
rs12000011	NTRK2	Intron variant	–	WTF1, Gfi1b
rs1948308	NTRK2	Intron variant	–	HIC2, Nkx3-1
rs3739570	NTRK2	3 prime UTR variant	–	–
rs10780695	NTRK2	Downstream gene variant	–	Stat2, HOXC13
rs2117655	NTRK3	Intron variant	–	SOX15, SOX8
rs11638486	NTRK3	Intron variant	–	–
rs1435403	NTRK3	Intron variant	–	Arid5a
rs922232	NTRK3	Intron variant	–	HOXC13
rs2009853	NTRK3	Intron variant	-	–
rs1461210	NTRK3	Intron variant	–	–
rs1346164	NTRK3	Intron variant	–	GFI1, Gfi1b
rs744993	NTRK3-AS1	Upstream gene variant	–	ZFP42
rs6328	NFG-AS1	Intron variant	–	INSM1
rs6537860	NFG-AS1	Intron variant	–	ZKSCAN5
rs1800601	NTRK1	5 prime UTR variant	Prom (EH38E1388452)	–
rs1998977	NTRK1	Intron variant	–	DMRTC2
rs10908521	NTRK1	Intron variant	–	–
rs1800879	NTRK1	Intron variant	–	RELB

First, we performed functional annotation of genetic variants based on ENCODE project data. Among the six SNPs that showed a significant p-value and thus appeared to be potentially associated with the presence of GD, two of them were associated with enhancers: rs6489630 at chromosome 12 that overlaps with a proximal enhancer-like signature downstream of NTF3 gene, and the intron variant rs11140783 at NTRK2 that is adjacent to another enhancer-like domain (see Table 1). However, four out of thirty non-significant SNPs were also associated with enhance-like landscapes (rs12273363, rs741073, rs4412435 and rs1800601 (Table 1).

By using JASPAR CORE, we identified several SNPs that overlap core consensus sequences for TF, and thus they could disrupt their binding or change their binding affinity (see Table 1). It is important to mention that rs7956189 located downstream of NTF3 gene overlaps the binding site of WT1, which has been associated with depressive-like behaviours in mice^41^. Another significant variant, rs3763614, in an intron of CNTFR, is placed in the binding site of NR5a2. This is a factor upregulated in the arcuate nucleus of the hypothalamus^42^. Rs11140783 located in an NTRK2 intron, could disrupt IRF8 binding, which has been proposed as a susceptibility factor for multiple sclerosis^43^ (see Table 1). Two significant variants rs3739570 (at 3’UTR of NTRK2) and rs10908521 (at an NTRK1 intron) did not show any association with regulatory regions or TF binding sequence consensus. Additionally, several non-significant variants overlaped with a variety of core TF binding sequences. Among them, stand out two SNPs that were associated with TF related to neuronal tissue. One is rs154528, another intron variant of NTRK2 which resides in the OTX2 binding site, a TF related with dopaminergic neurogenesis^44^. Importantly, genetic alterations in dopamine receptors 1 (DRD1) have been associated with disordered gambling^45^. Finally, rs1435403 overlaps the binding site of the Arid5a transcription factor, which has been associated with schizophrenia^46^.

Hi-C and micro-C data revealed interactions between different loci at the NTF3 gene region that expanded through the last exon and the 3′ UTR region. Intriguingly, the three GD-associated NTF3 SNPs (rs6332, rs6489630, rs7956189) showed interactions among them (see Fig. S1a at supplementary material). Similarly, rs3739570 and rs10780695 on the NTRK2 gene could also be interacting in the nucleus (see Fig. S1b at supplementary material).

## Discussion

The main aim of this study was to analyse the association between NTF genes and GD, as potential biological risk factors implicated in the aetiopathogenesis of GD. For this purpose, a strategy of pathway-based candidate genes was performed.

Both single and multiple-marker analysis showed a strong association between NTF3 and GD with a heterozygous disadvantage model of inheritance. NTF3 prevents the death of adult central noradrenergic neurons, promotes survival of ventral mesencephalic dopaminergic neurons, cerebellar granule neurons and Purkinje cells, and acts on sensory or sympathetic neurons of the dorsal root, nodose and sympathetic ganglia^47^. Moreover, NTF3 has been associated with affective disorders^48^ and schizophrenia^49^ and, as reported by Mercader, Saus et al.^50^ NTRK3 appears to be the gene with more association signals with eating disorders (EDs). In addition, enhanced neurogenesis caused by dietary restriction has been accompanied by increased NTF3 and BDNF levels^51^.

These results are worth noting because several studies have found that ED frequently co-occur with GD^52,53^ and have many commonalities considering psychological and clinical characteristics, such as personality traits and emotion regulation processes^39,54–56^. In particular, certain subtypes of EDs, such as bulimia nervosa (BN) or binge eating disorder (BED) have been associated with high levels of impulsivity^55,56^. Therefore, our findings may present an interesting unexplored genetic pathway underlying these disorders.

Comparison of the allele frequency distribution between groups indicated that the presence of G allele in NTF3 rs796189 is potentially associated with GD. Although this variant was located at a non-coding region, functional annotation based on ENCODE data showed that it is placed in a proximal enhancer-like signature, downstream of NTF3 gene. Transcriptional enhancer elements are non-coding stretches of DNA that have a central role in regulating transcriptional activity^57,58^. Interestingly, non-coding SNPs, that have been associated with risk for numerous common diseases through GWAS, frequently lie in these genomic elements, probably affecting their function^59^. Therefore, additional research is needed to delineate the impact of these variants on GD aetiology to enhance diagnosis, prevention strategies and treatment approaches.

By using JASPAR CORE we found that rs7956189 also overlapped the binding site of WT1, a TF associated with depressive-like behaviour in mice^41^. As proposed, the presence of a sequence variant within a binding site may alter the binding of the TF. As a consequence, gene expression, DNA methylation, and chromatin states would be altered too^60^. Although this variant did not imply a change in the coding sequences of NTF3, it appeared to be associated with changes in genomic function. Further epigenetic analysis, such as WT1 chromatin immunoprecipitation, is needed to understand its functional impact better. Haplotype analysis revealed an increased risk of GD in individuals carrying the G allele for rs7956189 who also carried the T allele of rs6489630 in NTF3 gene. Curiously, the T allele of rs6489630 has been recently associated with significantly lower intelligence scores than C allele carriers in children with Attention-Deficit/Hyperactivity Disorder (ADHD)^61^. However, a recent study suggested a protective role of this allele against Alzheimer disease^62^. Thus, it has been proposed that the rs6489630 polymorphism might have age-related influences.

Interestingly, some authors described ADHD in childhood as a risk factor for the development of addictive behaviours^63^, including GD^64^. In addition, the association between ADHD and GD is noteworthy with approximately 25% of adults with GD meeting ADHD criteria^65^. In particular, the hyperactive subtype of ADHD is a developmental disorder characterised by impulsivity that commences in childhood and is often observed in conduct disorder and antisocial personality disorder^10^. Indeed, as proposed by the pathways model of GD, the third subgroup of addicted gamblers, is differentiated by features of impulsivity and antisocial personality disorder and attention deficit^16^. In this vein, our group examined the association between ADHD symptomatology, emotion regulation and GD^66^. It showed greater emotional dysregulation and GD severity among patients with GD who had ADHD symptoms. Hence, T allele of rs6489630 would be potentially associated with the presence of GD. Future research may consider underlying genetic features common to both ADHD and GD.

Single marker analysis has also shown a strong association between CNTFR rs3763614 variant and GD, with a heterozygous advantage model of inheritance. Although this model of inheritance has received little attention, up to 50% of all gene associations in psychiatric disorders have been shown to occur under a heterozygous advantage^67,68^.

At a molecular level, CNTF (i.e. CTNFR ligand) showed to have an important effect on appetite and energy expenditure^69,70^. This leptin-like molecule activates signalling cascades in the hypothalamic nuclei involved in feeding control^71–73^. Our findings, in line with previous studies, suggest the existence of a common genetic pathway that could validate the NTF hypothesis role in some disorders related to impulsivity^50^.

Both single and multiple-marker analysis also showed a strong association between NTRK2 and GD, specifically with two genetic variants, namely rs11140783 and rs3739570. Moreover, a haplotype block was manually defined for these two SNPs, showing a significant association of haplotype CC with the risk of GD. As several studies have shown that both BDNF and NTRK2 (i.e., BDNF receptor) are involved in the regulation of eating behaviours and energy balance^74,75^, it is not surprising that this receptor appears to be associated with GD. Engagingly, NTRK2 can also alternatively bind NTF3, also showing to be significantly associated with GD. Although this factor is less efficient in activating NTRK2, it appears to regulate neuron survival through this receptor^76,77^. Again, these results support the idea of a common genomic pathway that may help to explain the aetiopathogenesis of impulse control disorders, such as EDs and GD.

The analysis of the 3D structure of chromatin inside the nucleus has revealed the existence of hierarchical chromatin organization; compartments, topologically associating domains (TADs), sub-TADs, insulated domains and chromatin loops. The importance of the spatial organization of DNA for transcriptional regulation is now widely accepted^78–80^. We used publicly available Hi-C data to explore spatial proximity of our distant genetic variants. At an intragenic level, we found a slight interaction between variants rs6332, rs6489630 and rs7956189 located in the NTF3 gene. Similarly, rs3739570 and rs10780695 on the NTRK2 gene could be also interacting in the nucleus. Although phenotypic consequences of the interactions’ disruption and domains emerging from Hi-C experiments are still controversial, several works showed extremely subtle changes in contact frequencies associated with large differences in gene expression^81^. Whether this could be the case for the NTFs loci discussed here remains to be addressed.

A couple of final considerations on Hi-C data would be that it was obtained from human embryonic stem cells (hESC), highlighting the importance of these interactions before the neuronal lineage is specified. Moreover, as chromatin contacts are dynamic, we can expect a different scenario when analysing it in tissues more relevant to GD such as the neuronal lineages. Nevertheless, the data gathered here strongly suggests the existence of a possible relationship between genetic and epigenetic mechanisms implicated in GD aetiopathogenesis.

Taken all together, our findings suggest the involvement of different members of the NTF signalling pathway, and support the possibility that GD could be the result of an altered cross-regulation of the different NTFs, as was previously proposed by Mercader et al.^50^ regarding EDs. Indeed, a considerable crosstalk among intracellular signaling pathways of NTFs has been previously reported^82^. So, genetic variants affecting the expression of several NTF genes may alter biochemical interactions between ligands, their receptors and their intracellular target proteins, that eventually alter neuronal functions. Moreover, results obtained in the present work strongly support the existence of common biological mechanisms underlying several psychiatric disorders, which reinforce the idea of shared core transdiagnostic features, such as impulsivity. This is in agreement with the three pathways model of GD proposed by Blaszczynski and Nower^16^, that integrates the complex array of biological, psychological and sociodemographic determinants of GD. However, further studies are needed to better understand the exact role of NTFs, and its implication in specific psychological processes.

The presence of a control group and the wide genetic analysis of the NTF genes variants are highlighted as part of the strength of this work. Nevertheless, this study should be also seen considering some limitations, such as bias due to the self-reported data in the clinical assessment or a modest sample size. We have computed statistical power for the comparison of proportions for a total sample size of N = 357 with a n-ratio equal to 191/166 = 1.15, an alpha value equal to a = 0.05 and different values of delta differences (d): (a) for identifying values of d = 0.15 (differences of at least 15%), the power is 0.8182 (above the cut-off usually recommended as a reasonable balance between Type I and Type II errors for research studies; (b) for identifying values of d = 0.10 (differences of at least 10%), the power is decreased to 0.4722. Therefore, some of the comparisons of this work were underpowered, and effects of practical importance could be not detected. Finally, in future studies, including larger samples and the functional impact of selective genetic variants on the GD aetiopathology should be evaluated.

In conclusion, the present study suggests the involvement of different NTF and its receptors in GD aetiopathogenesis. Moreover, it proposes the possibility that genetic predisposition to GD may be the result of an altered cross-regulation of different members of the NTF signalling pathway. Our findings provide further evidence regarding the existence of an interesting genetic and epigenetic pathway that could validate the NTF hypothesis role in the development of psychiatric disorders^83,84^.

## Methods

### Participants

Entry into the study was between January 2005 and June 2006. The sample included a clinical group of *n* = 166 patients with GD consecutively admitted to the Outpatient GD Unit in the Department of Psychiatry at the University Hospital of Bellvitge. All the subjects of this group fulfilled the DSM-IV criteria for GD as determined by a face-to-face semi-structured clinical interview (SCID-I)^85,86^. The control group included *n* = 191 participants, recruited via advertisement in the catchment area. Table 2 includes groups description.

**Table 2:** Description of the samples.

Sociodemographic variables	Control (*n* = 191)		GD (*n* = 166)		*p*	GD related variables	GD (*n* = 166)	
	n	%	n	%			Mean	SD
**Sex**								
Women	113	59.2%	14	8.4%	< 0.001	Age of onset of GD	33.8	11.6
Men	78	40.8%	152	91.6%		Duration of GD	13.6	8.3
**Education**								
Primary	41	21.5%	120	72.3%	< 0.001	DSM-5 criteria for GD	7.2	2.1
Secondary	35	18.3%	39	23.5%		Gambling activities	n	%
University	115	60.2%	7	4.2%		Slot-machines	149	89.8%
**Employment**								
Unemployed	6	3.1%	48	28.9%	< 0.001	Bingo	35	21.1%
Employed	185	96.9%	118	71.1%		Lotteries	13	7.8%
	Mean	SD	Mean	SD		Casino	17	10.2%
Age (years-old)	42.4	11.7	39.9	12.4	0.049	Cards	11	6.6%

*SD* standard deviation.

### Clinical assessment and procedure

*Diagnostic Questionnaire for Pathological Gambling According to DSM criteria*^87^: a 19 items self-report questionnaire used for diagnosing GD according to DSM-IV-TR^86^. The Spanish version of the questionnaire obtained satisfactory psychometric properties (Cronbach’s alpha *α* = 227 0.81 for a population-based sample and *α* = 0.77 for a clinical sample)^88^. Additional socio-demographic and clinical information (e.g. age of onset, duration of GD) were also assessed using a semi-structured face-to-face clinical interview described elsewhere^89^.

### SNP genotyping

Single nucleotide polymorphisms (SNPs) of several NTF genes [nerve growth factor (NGF) gene and its receptor (NGFR), neurotrophic tyrosine kinase receptor type 1 (NTRK1), type 2 (NTRK2) and type 3 (NTRK3), BDNF, NTF3, NTF4, ciliary neurotrophic factor (CNTF), and its receptor (CNTFR)] were selected and genotyped as previously described by Mercader, Saus et al.^50^. Only SNPs were considered for further analysis, as they have a unique mapping location on the NCBI B34 assembly and a minor allele frequency (MAF) higher than 10%. Bins of common SNPs in strong LD, as defined by R2 higher than 0.85, were identified within this data set by using HapMap-LDSelect-Processor, which uses the ‘LD Select’ method to process HapMap genotype dump format data corresponding to the locus region for each gene, covering the entire gene and 10 kb upstream and downstream the gene. One hundred and eighty-three TagSNPs were selected for coverage of all bins for a total of 10 genes.

Next, Tag-SNPs were genotyped using the SNPlex Genotyping System (Applied Biosystems, Foster City, CA, USA) at the genotyping facilities of CeGen, in the Barcelona Node (Centro Nacional de Genotipado, Genoma España). To examine the reliability of the new genotyping and the coincidence of genotype calls between the two time-independent projects, the new batch included the same DNA control samples used in Mercader, Saus et al.^50^ namely six samples corresponding to two HapMap reference trios: samples NA10860, NA10861, NA11992, NA11993, NA11994 and NA11995 (family numbers CEPH131 and CEPH132). Both genotype concordance and correct Mendelian inheritance of these samples in the two different batches was verified using SNPator (http://www.cegen.org). Of the whole available sample, genotyped SNPs, which had a call rate lower than 80%, were outside Hardy Weinberg equilibrium (HWE), or were monomorphic and not considered for the analyses (n = 25).

### Statistical analyses

Haploview4.2 for Windows^90^ was used to analyse the LD and haplotype construction, as well as the risk association between the SNPs (single markers) and the haplotypes with GD diagnosis. In this study, the default parameters of the program were used: cut-off equal to 0.001 for the Hardy–Weinberg equilibrium test (HWE), minimum genotype 75% and minimum minor allele frequency equal to 0.001. The linkage case–control format was defined for the data file, and the haplotype block recognition was based on Gabriel’s algorithm^91^, which uses the Hedrick’s multiallelic *D’* to measure the degree of LD between the blocks (considering each haplotype within a block as an “allele” of that region) (the coefficient *D’* goes into the range − 1 to + 1: *D*′ = 0 indicates that the two markers are independent [perfect equilibrium] and *D*′ = 1 indicates that no more than 3 of the 4 possible haplotypes are being observed in the sample [complete disequilibrium]).

Stata16 for Windows was used to perform other statistical analysis. T-test procedures compared means of data, and chi-square test (χ^2^) and logistic regressions compared proportions of data and assessed the discriminative capacity of the SNPs to identify the presence of GD. Due to the multiple null-hypothesis tests, increase in Type-I error was controlled with Simes’ correction method, based on the ordered *p*-values, less conservative than the classical Bonferroni procedure and particularly useful when several correlated test statistics are involved^92^.

### Exploratory functional element analysis

The identification of regulatory elements overlapping the identified SNPs variants associated to GD was performed using SCREEN, a web interface for searching and visualizing the Registry of candidate cis-Regulatory Elements (cCREs) derived from ENCODE data^93^.

The putative effect of variants analysed on TF binding motifs were computationally analysed via JASPAR. It is the largest open access database of matrix-based nucleotide profiles describing the binding preference of transcription factors from multiple species. JASPAR CORE database (http://jaspar.genereg.net/, version 5.0) contains a curated and non-redundant set of profiles derived from published collections of experimentally defined transcription factor binding sites for eukaryotes^94^. Here, we used the UCSC Genome Browser Track Hub that represents genome-wide predicted binding sites for TF binding profiles in the JASPAR database CORE collection. We then assessed whether the SNPs analysed were overlapping core consensus TF binding sequences and as such, they could potentially disrupt the binding of transcription factors or change their binding affinity. Only human matrix models were selected, and motifs with JASPAR score 5 were listed.

Finally, UCSC Genome Browser Hi-C and Micro-C Track Search feature (http://genome.ucsc.edu/) was used to explore potential interactions of heatmaps of chromatin folding between our candidate genetic variants. A high score between two regions suggests that they probably interact, or they are in proximity in 3D space within the nucleus of a cell. In the track display, this is shown by a more intense colour in the heatmap, or by an arch.

### Ethics

The study procedures were carried out in accordance with the Declaration of Helsinki. The University Hospital of Bellvitge Ethics Committee of Clinical Research approved the study (reference: 307/06). All the participants provided voluntary-signed informed consent and received no financial remuneration. Psychometric assessments were carried out by experienced psychologists and psychiatrists in the field of GD, at the Outpatient GD Unit.

## Supplementary Information

Supplementary Information.

## Data Availability

The genetic data analysed in the present project is part of a clinical database and cannot be shared publicly. In their informed consent, patients signed a document providing their genetic and clinical data to the hospital. For this reason, all this information is stored in a repository at the hospital’s research center (datamanagement@idibell.cat). However, it would be available upon contact with the corresponding author.
