# Supplementary material for: The role of neurotrophin genes involved in the vulnerability to gambling disorder
Source: Sci Rep. 2022 Apr 28;12:6925. doi: 10.1038/s41598-022-10391-w (PMC9051155; doi:10.1038/s41598-022-10391-w)

Table S1. Distribution of allele frequencies of the studied SNPs and association for single markers

Marker				HWE	Association test (single markers)				
SNP	Chromosome	Gene	Alleles	*p*	Allele	CCR counts	CCR frequencies	$|^2$	*p*
rs6332	12:5494466	NTF3	A:G	0.632	G	160:172, 180:194	0.482, 0.481	0	0.986
rs6489630	12:5495458	NTF3	C:T	1	T	75:251, 63:309	0.230, 0.169	4.037	**0.045***
rs7956189	12:5496695	---	A:G	0.505	G	56:264, 42:312	0.175, 0.119	4.296	**0.038***
rs12273363	11:27723312	BDNF	T:C	0.567	T	275:49, 294:62	0.849, 0.826	0.653	0.419
rs908867	11:27724217	---	G:A	1	G	309:21, 333:39	0.936, 0.895	3.798	0.051
rs1491850	11:27728178	---	T:C	0.876	T	206:126, 216:148	0.620, 0.593	0.533	0.465
rs2274592	9:34552439	CNTFR	T:C	0.279	T	239:85, 258:102	0.738, 0.717	0.378	0.539
rs4363285	9:34553669	CNTFR	C:T	0.992	C	278:44, 279:63	0.863, 0.816	2.776	0.096
rs10814123	9:34561810	CNTFR	C:T	0.641	C	290:36, 276:50	0.890, 0.847	2.625	0.105
rs3763614	9:34584137	CNTFR	C:T	1	C	308:10, 347:27	0.969, 0.928	5.638	**0.018***
rs657770	17:49503788	NGFR	C:A	0.687	A	131:201, 112:222	0.395, 0.335	2.522	0.112
rs534561	17:49508850	NGFR	C:G	0.351	G	126:206, 112:222	0.380, 0.335	1.416	0.234
rs741073	17:49514524	NGFR	G:A	0.772	G	256:76, 256:96	0.771, 0.727	1.742	0.187
rs11140783	9:84839902	NTRK2	C:T	0.477	C	316:14, 344:30	0.958, 0.920	4.273	**0.039***
rs1545285	9:84927454	NTRK2	C:A	1	C	178:148, 204:172	0.546, 0.543	0.008	0.927
rs4412435	9:84970582	NTRK2	T:C	0.261	T	205:111, 215:145	0.649, 0.597	1.898	0.168
rs10868241	9:84978113	NTRK2	G:A	1	G	255:75, 269:105	0.773, 0.719	2.634	0.105
rs4361832	9:84980819	NTRK2	G:A	1	G	278:50, 312:58	0.848, 0.843	0.025	0.875
rs12000011	9:84991960	NTRK2	C:T	0.549	C	247:75, 263:105	0.767, 0.715	2.446	0.118
rs1948308	9:85001342	NTRK2	T:C	1	T	203:121, 213:155	0.627, 0.579	1.638	0.201
rs3739570	9:85022663	NTRK2	C:T	0.717	C	308:18, 337:37	0.945, 0.901	4.598	**0.032***
rs10780695	9:85029318	NTRK2	C:T	0.413	C	245:71, 272:86	0.775, 0.760	0.227	0.634
rs2117655	15:87885471	NTRK3	T:G	0.297	G	129:201, 138:228	0.391, 0.377	0.141	0.707
rs11638486	15:87888940	NTRK3	C:T	0.004	T	98:224, 90:246	0.304, 0.268	1.073	0.3
rs1435403	15:87893838	NTRK3	C:T	0.024	T	96:234, 94:276	0.291, 0.254	1.198	0.274
rs922232	15:87971845	NTRK3	A:T	0.64	A	208:122, 224:150	0.630, 0.599	0.728	0.394
rs2009853	15:87986102	NTRK3	T:A	0.816	A	61:267, 57:309	0.186, 0.156	1.121	0.29
rs1461210	15:88085748	NTRK3	G:C	0.698	G	170:160, 187:177	0.515, 0.514	0.001	0.97
rs1346164	15:88186248	NTRK3	G:C	0.191	G	224:82, 252:118	0.732, 0.681	2.087	0.149
rs744993	15:88260034	NTRK3-AS1	G:T	0.401	G	289:35, 324:48	0.892, 0.871	0.728	0.394
rs6328	1:115287322	NGF-AS1	G:T	0.39	G	247:85, 235:109	0.744, 0.683	3.056	0.081
rs6537860	1:115313723	NGF-AS1	G:A	0.33	G	256:76, 240:100	0.771, 0.706	3.694	0.055
rs1800601	1:156815825	NTRK1	T:C	0.691	C	118:212, 99:243	0.358, 0.289	3.563	0.059
rs1998977	1:156834329	NTRK1	C:T	0.146	T	119:209, 112:234	0.363, 0.324	1.143	0.285
rs10908521	1:156843858	NTRK1	T:C	0.67	C	100:232, 81:265	0.301, 0.234	3.898	**0.048***
rs1800879	1:156868349	NTRK1	C:T	0.455	T	124:182, 112:222	0.405, 0.335	3.352	0.067

Note. HWE: Hardy-Weinberg equilibrium test. CCR:Case,control ratios. *Bold: significant association (0.05).

Table S2 Single nucleotide polymorphism under different genetic models and assessment of risk for the presence of GD

		Codominant						Dominant				Recessive				Overdominant				Log-add.
rs6489630		CC		CT		TT		CC		CT/TT		CC/CT		TT		CC/TT		CT		*p* = .266
Control	n-%	128	68.8%	53	28.5%	5	2.7%	128	68.8%	58	31.2%	181	97.3%	5	2.7%	133	71.5%	53	28.5%	
GD	n-%	96	58.9%	59	36.2%	8	4.9%	96	58.9%	67	41.1%	155	95.1%	8	4.9%	104	63.8%	59	36.2%	
	p-value	.266						.168				.222				.372				
rs7956189		AA		AG		GG		AA		AG/GG		AA/AG		GG		AA/GG		AG		*p* = .169
Control	n-%	138	77.5%	38	21.3%	2	1.1%	138	77.5%	40	22.5%	176	98.9%	2	1.1%	140	78.7%	38	21.3%	
GD	n-%	107	66.9%	50	31.3%	3	1.9%	107	66.9%	53	33.1%	157	98.1%	3	1.9%	110	68.8%	50	31.3%	
	p-value	.169						**.049***				.730				**.048***				
rs3763614		CC		CT		TT		CC		CT/TT		CC/CT		TT		CC/TT		CT		*p* = .054
Control	n-%	161	86.1%	25	13.4%	1	0.5%	161	86.1%	26	13.9%	186	99.5%	1	0.5%	162	86.6%	25	13.4%	
GD	n-%	149	93.7%	10	6.3%	0	0%	149	93.7%	10	6.3%	159	100%	0	0%	149	93.7%	10	6.3%	
	p-value	**.049***						**.024***				.999				**.036***				
rs11140783		CC		CT		--		--		--		--		--		--		--		---
Control	n-%	157	84.0%	30	16.0%															
GD	n-%	151	91.5%	14	8.5%															
	p-value	**.006***																		
rs3739570		CC		CT		TT		CC		CT/TT		CC/CT		TT		CC/TT		CT		*p* = .073
Control	n-%	152	81.3%	33	17.6%	2	1.1%	152	81.3%	35	18.7%	185	98.9%	2	1.1%	154	82.4%	33	17.6%	
GD	n-%	146	89.6%	16	9.8%	1	0.6%	146	89.6%	17	10.4%	162	99.4%	1	0.6%	147	90.2%	16	9.8%	
	p-value	.097						**.043***				.608				**.048***				
rs10908521		TT		TC		CC		TT		TC/CC		TT/TC		CC		TT/CC		TC		*p* = .195
Control	n-%	100	57.7%	67	38.5%	7	4.0%	100	57.5%	74	42.5%	167	96.0%	7	4.0%	107	61.5%	67	38.5%	
GD	n-%	81	48.8%	70	42.2%	15	9.0%	81	48.8%	85	51.2%	151	91.0%	15	9.0%	96	57.8%	70	42.2%	
	p-value	.511						.249				.648				.354				

Note. *Bold: significant association (.05). *p-values obtained in logistic regression adjusted by sex-age-education-employment*

Table S3. Haplotype frequencies and association test

Block	SNPs	Gene	Haplotype	Frequency	CCR counts	CCR frequencies	χ^2	*p*	Block-joint test
Block 1	rs6489630	NTF3	CA	0.801	254.7 : 75.3, 310.7 : 65.3	0.772, 0.826	3.293	0.105	χ^2=5.58; *p*=**.045***
	rs7956189		TG	0.14	56.7 : 273.3, 42.4 : 333.6	0.172, 0.113	5.075	**0.044***	
			TA	0.057	18.6 : 311.4, 21.8 : 354.2	0.056, 0.058	0.009	0.925	
Block 2	rs2274592	CNTFR	TC	0.723	239.9 : 86.1, 260.6 : 105.4	0.736, 0.712	0.486	0.486	χ^2=2.93; *p*=.222
	rs4363285		CT	0.166	45.8 : 280.2, 69.0 : 297.0	0.140, 0.189	2.874	0.270	
			CC	0.111	40.3 : 285.7, 36.4 : 329.6	0.124, 0.099	1.029	0.465	
Block 3	rs4412435	NTRK2	TGG	0.617	212.8 : 119.2, 225.3 : 152.7	0.641, 0.596	1.507	0.440	χ^2=5.61; *p*=.174
	rs10868241		CAA	0.156	51.8 : 280.2, 59.2 : 318.8	0.156, 0.157	0.000	0.982	
	rs4361832		CGG	0.127	43.2 : 288.8, 46.8 : 331.2	0.130, 0.124	0.062	0.885	
			CAG	0.095	22.0 : 310.0, 45.2 : 332.8	0.066, 0.120	5.906	**0.048***	
Block 4	rs12000011	NTRK2	CT	0.603	207.9 : 122.1, 220.4 : 159.6	0.630, 0.580	1.853	0.251	χ^2=3.06; *p*=.380
	rs1948308		TC	0.264	78.3 : 251.7, 109.0 : 271.0	0.237, 0.287	2.225	0.231	
			CC	0.133	43.8 : 286.2, 50.6 : 329.4	0.133, 0.133	0.001	0.980	
Block 5	rs211765	NTRK3	TCC	0.614	200.7 : 131.3, 236.6 : 143.4	0.604, 0.623	0.248	0.999	χ^2=1.88; *p*=.599
	rs11638486		GTT	0.269	94.9 : 237.1, 96.4 : 283.6	0.286, 0.254	0.924	0.618	
	rs1435403		GCC	0.092	28.1 : 303.9, 37.1 : 342.9	0.085, 0.098	0.367	0.806	
			GTC	0.022	6.3 : 325.7, 9.7 : 370.3	0.019, 0.025	0.337	0.806	
Block 6	rs922232	NTRK3	AT	0.445	147.7 : 184.3, 169.0 : 211.0	0.445, 0.445	0.000	0.998	χ^2=2.05; *p*=.432
	rs2009853		TT	0.386	122.5 : 209.5, 152.3 : 227.7	0.369, 0.401	0.765	0.591	
			AA	0.169	61.8 : 270.2, 58.7 : 321.3	0.186, 0.154	1.281	0.573	
[1]Block 7	rs11140783	NTRK2	CC	0.867	299.1 : 30.9, 311.2 : 62.8	0.906, 0.832	8.345	**0.012***	χ^2=10.07; *p*=**.001***
	rs3739570		CT	0.071	16.9 : 313.1, 32.8 : 341.2	0.051, 0.088	3.525	0.072	
			TC	0.055	12.6 : 317.4, 25.8 : 348.2	0.038, 0.069	3.228	0.072	

Note. CCR:Case,control ratios. *Bold: significant association (.05). [1]Block manually defined for rs11140783 and rs3739570

Figure S1. Hi-C interaction map for a) rs6332, rs6489630 and rs7956189 of NTF3 gene *and b) rs3739570 and rs10780695 on the NTRK2 gene*

a)

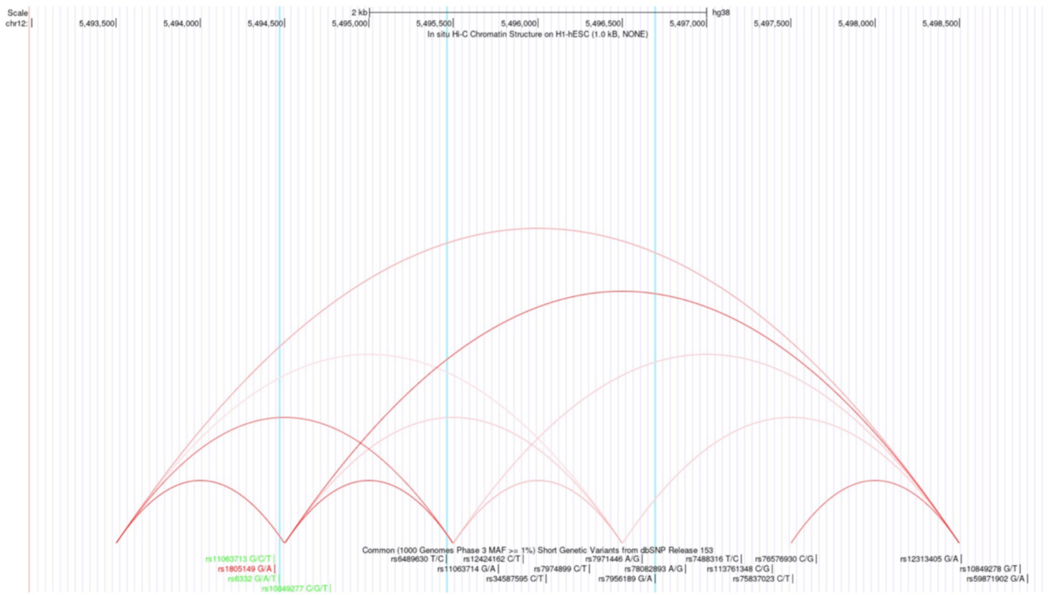

b)

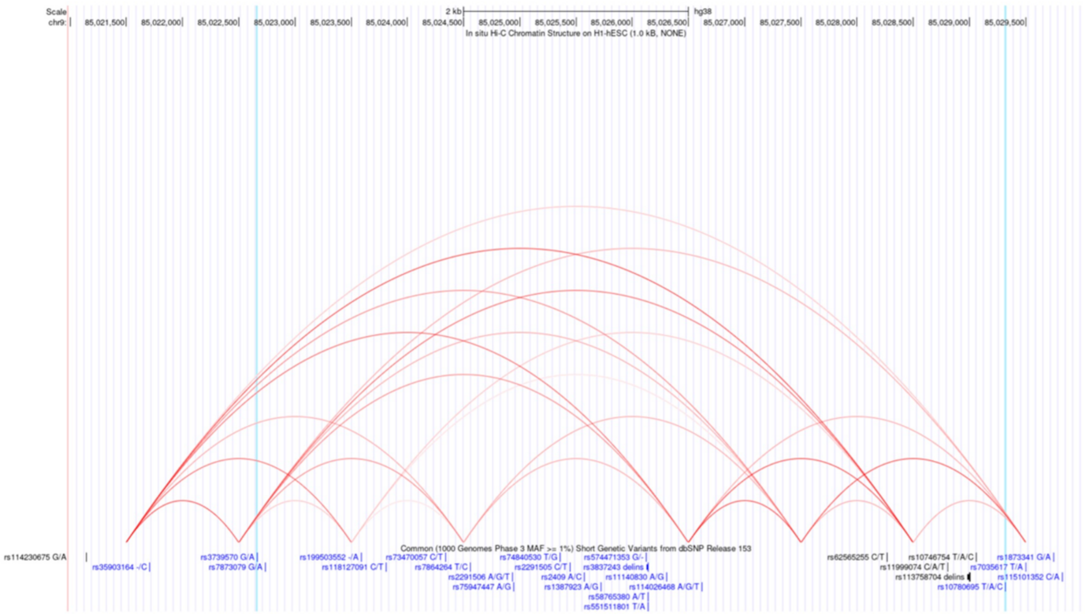

Supplement: Supplementary file 1 — Supplementary Information. [file 41598_2022_10391_MOESM1_ESM.pdf]
